# Magnetically Guided Capsule Endoscopy in Pediatric Patients with Abdominal Pain

**DOI:** 10.1155/2019/7172930

**Published:** 2019-05-08

**Authors:** Mingping Xie, Yuting Qian, Shidan Cheng, Lifu Wang, Ruizhe Shen

**Affiliations:** Department of Gastroenterology, Ruijin Hospital Affiliated to Shanghai Jiao Tong University School of Medicine, Shanghai 200025, China

## Abstract

**Background and Aims:**

Magnetically guided capsule endoscopy (MGCE) offers a noninvasive method of evaluating both the gastric cavity and small intestine; however, few studies have evaluated MGCE in pediatric patients. We investigated the diagnostic efficacy of MGCE in pediatric patients with abdominal pain.

**Patients and Methods:**

We enrolled 48 patients with abdominal pain aged 6–18 years. All patients underwent MGCE to evaluate the gastric cavity and small intestine.

**Results:**

The cleanliness of the gastric cardia, fundus, body, angle, antrum, and pylorus was assessed satisfactorily in 100%, 85.4%, 89.6%, 100%, 97.9%, and 100% of patients, respectively. The subjective percentage visualization of the gastric cardia, fundus, body, angle, antrum, and pylorus was 84.8%, 83.8%, 88.5%, 87.7%, 95.2%, and 99.6%, respectively. Eighteen (37.5%) patients had 19 gastrointestinal tract lesions: one esophageal, three in the gastric cavity, and 15 in the small intestine. No adverse events occurred during follow-up.

**Conclusions:**

MGCE is safe, convenient, and tolerable for evaluating the gastric cavity and small intestine in pediatric patients. MGCE can effectively diagnose pediatric patients with abdominal pain.

## 1. Introduction

Capsule endoscopy was first used in 2001 and is now widely accepted. Capsule endoscopy is a noninvasive method of evaluating the small intestine, which is critical to diagnose small intestinal disease [1, 2]; however, the procedure has a limited role when examining the gastric cavity because of the stomach's unique anatomy. Using an external magnetic field, magnetically guided capsule endoscopy (MGCE) provides evaluation of the complete gastric cavity [3]. MGCE is much more tolerable than traditional gastroscopy for patients and can avoid adverse reactions and discomfort caused by anesthesia and surgery. Several studies have shown that MGCE provides complete visualization of all parts of the stomach [4–7] and has similar accuracy and specificity compared with traditional gastroscopy [6–10]. However, few studies have evaluated MGCE in pediatric patients.

Abdominal pain is a common complaint of patients visiting a gastroenterology department, and patients often require examination of the gastric cavity and possibly the small intestine. We investigated the diagnostic efficacy of MGCE to evaluate both the gastric cavity and small intestine in pediatric patients with abdominal pain.

## 2. Patients and Methods

### 2.1. Patients

We enrolled patients aged 6–18 years of age with a complaint of abdominal pain to undergo MGCE examination between January 2017 and October 2018. We excluded the following: patients with dysphagia, suspected or known gastrointestinal stenoses or obstruction, or congenital gastrointestinal malformations or intestinal fistula; patients in poor general condition and unable to tolerate the examination; and patients with pacemakers, defibrillators, or other implants that could be affected by external magnetic fields. All patients and their guardians provided informed consent, and the study was approved by the Ruijin Hospital Ethics Committee.

### 2.2. MGCE System

We used the system manufactured by Ankon Technologies Co. Ltd. (Wuhan, China), which was approved by the China Food and Drug Administration in 2013. The system consists of capsules, a control system, a portable recorder, and a capsule locator. The capsule weights 5 g and has a diameter of only 11.8 mm × 27 mm. The capsule has a built-in camera, wireless transceiver, and four light-emitting diodes and magnet, all of which are sealed in a capsule made of biocompatible material. The camera takes two pictures per second and has a viewing angle of 140 degrees. The control system consists of a translational rotary table, a bed, a magnetic head, two monitors, and a console. By adjusting the movement of the magnetic head and generating a corresponding magnetic field, we can control the movement of the capsule within the body. A portable recorder is contained in a suit, which is easy to wear, and adjustable buckles allow for an individualized fit for the patient. Rechargeable lithium batteries provide more than 8 hours of working time. The capsule locator can detect the capsule and can confirm that the capsule has been excreted. This technique is safe and nonradiative.

### 2.3. Procedures

Gastrointestinal preparation began at 8 pm the day before examination. For patients older than 10 years or weighing >40 kg, 2000 mL of polyethylene glycol (PEG) solution was administered as for adults. Patients ≤10 years old or weighing ≤40 kg received 25 mL/kg of PEG. Oliva et al. [11] showed that the use of low PEG volumes (25 mL/kg) did not affect the quality of bowel preparation compared with high PEG volumes (50 mL/kg) in pediatric patients. All patients fasted overnight, and 60 minutes before capsule ingestion, patients received 10 mL (400 mg) of simethicone emulsion and 200 mL of clear water. These were followed with another 300–500 mL of clear water, 15–30 minutes before capsule ingestion.

We recorded the following patient information: age, sex, weight, and indications for MGCE. Wearing the portable recorder, patients swallowed the capsule. Lying on the bed, patients were asked to change position from left lateral, to supine, to right lateral, then sitting, if necessary. During the examination, some patients required additional water to extend the gastric cavity. An experienced examiner controlled the capsule to evaluate the gastric cavity and, if possible, the bulb and descending duodenum. Following the gastric cavity examination, patients continued to wear the portable recorder for more than 7 hours to permit evaluation of the small intestine. We asked all patients whether they were willing to undergo the examination again. Additionally, for those who had undergone conventional gastroscopy, we asked which examination they preferred. All patients were followed for 2 weeks to record adverse events and to confirm capsule excretion.

**Figure 1:**
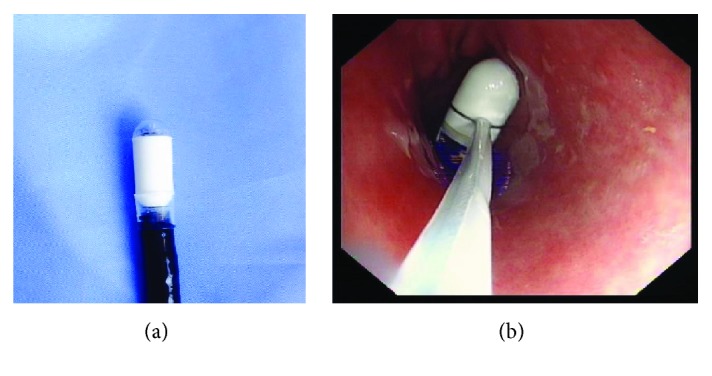
(a) Transparent hood-assisted endoscopic delivery (capsule endoscope loaded on the tip of gastroscope by a transparent hood); (b) endoscopic snare loop.

For patients who failed to swallow the capsule, a transparent hood-assisted endoscopic delivery device was used to deliver the capsule into the esophagus. When the capsule remained in the stomach for >1.5 hours, we added gastric motility-promoting drugs. If the capsule still failed to pass through the pylorus, we used an endoscopic snare loop to deliver the capsule to the duodenum (Figure 1).

### 2.4. Statistical Analysis

Continuous data were summarized as mean and standard error, mean and range, or median and range, and categorical data were presented as proportions. Comparisons between groups were performed using Student's *t* test, Mann-Whitney *U* test or the chi-squared test. *p* < 0.05 was considered statistically significant. All statistical analyses were performed using SPSS version 22.0 (IBM Inc., Armonk, NY, USA).

## 3. Results

### 3.1. Patients

We enrolled 48 patients: 32 (66.7%) boys and 16 (33.3%) girls with a mean age of 12.0 ± 2.8 years (range, 7–17 years). All patients successfully swallowed the capsule without the assistance of the endoscopic delivery device. Twenty-nine (60.4%) patients complained of abdominal pain only, while 19 (39.6%) patients had other complaints (six with vomiting, seven with digestive gastrointestinal bleeding, three with diarrhea and gastrointestinal bleeding, one with diarrhea and oral ulcers, one with oral ulcers, and one with skin rash).

### 3.2. Transit Times

The mean gastric evaluation time, which was defined as the time the examiner manipulated the capsule, was 8.5 minutes (range, 5–17 minutes). The mean gastric transit time, defined as the time the capsule remained in the gastric cavity, was 54 minutes (range, 5–254 minutes), and the mean small intestinal transit time was 246 minutes (range, 86–561 minutes). In nine (18.6%) patients, the examiner controlled the capsule passing through the pylorus to detect the bulb and descending duodenum, but in two patients, gastric motility-promoting drugs, namely, metoclopramide, 2.5–5 mg by intramuscular injection, were used, and in 1 patient, both gastric motility-promoting drugs and the endoscopic snare loop were used to deliver the capsule into the duodenum. At the end of the examination, the capsule had not passed the ileocecal valve in three patients.

### 3.3. Gastric Cleanliness and Mucosal Visualization

We assessed the cleanliness of the gastric cavity as excellent (gastric mucosa appearing clear and almost no bubbles or mucus affecting the field of vision; score, 100%), good (a small amount of bubbles or mucus affecting the field of vision; score, 75%), fair (moderate amount of bubbles or mucus affecting the field of vision; score, 50%), poor (a large amount of bubbles or mucus affecting the field of vision; score, 25%). In which, excellent and fair were satisfactory. The median cleanliness of the gastric cardia, fundus, body, angle, antrum, and pylorus was 100 (75, 100) %, 75 (50, 100) %, 75 (50, 100) %, 100 (75, 100) %, 100 (50, 100) %, and 100 (75, 100) %, respectively. And the cleanliness of the gastric cardia, fundus, body, angle, antrum, and pylorus was assessed satisfactorily in 100%, 85.4%, 89.6%, 100%, 97.9%, and 100% of patients, respectively. In general, the cleanliness of the distal cavity was better than in the proximal cavity. Statistically, the cleanliness of the cardia, angle, antrum, and pylorus was significantly better than that of the fundus and body (*p* < 0.05).

We also subjectively assessed the proportion of visible mucosa as a range from 0% to 100%. The percentage of visible mucosa in the gastric cardia, fundus, body, angle, antrum, and pylorus was 84.8%, 83.8%, 88.5%, 87.7%, 95.2%, and 99.6%, respectively, with better visualization distally compared with proximally generally. Statistically, the visualization of pylorus was significantly better than the others (*p* < 0.05), followed by the antrum, which was also significantly better than that of the fundus, body, and angle (*p* < 0.05). In one patient, excessive gastric emptying motility pushed the capsule into the duodenum before complete examination of the stomach was possible.

### 3.4. Diagnostic Yield

**Figure 2:**
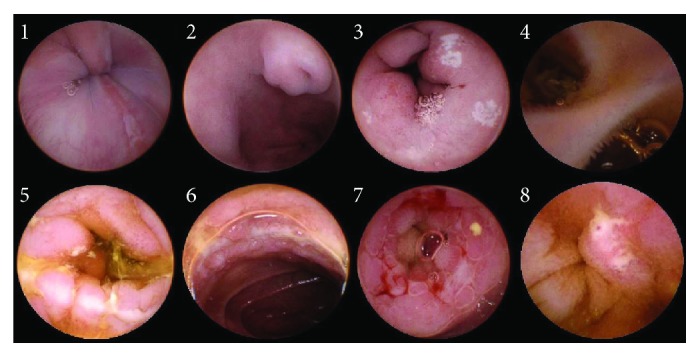
MGCE findings. 1: reflux esophagitis; 2: ectopic pancreas; 3: duodenal ulcers; 4: diverticulum; 5-8: Crohn's disease.

We found 19 gastrointestinal tract lesions in 18 patients (37.5%), although some of the lesions might have been unrelated to the patients' abdominal pain. One patient (2.1%) had an erosion above the dentate line and was finally diagnosed with reflux esophagitis. Lesions were detected in the stomach in three patients (6.3%), namely, a polyp, a protuberant lesion, and congestion and edema. These three patients were finally diagnosed as having a gastric polyp, ectopic pancreas, and congestive exudative gastritis, respectively. Fifteen patients (23.0%) had lesions in the small intestine including four with duodenal ulcers. Eight patients had ulcers in the jejunoileum: two patients had ulcers in the jejunum only, two in the ileum only, and four in both the jejunum and ileum; six of these eight patients were eventually diagnosed with Crohn's disease. The patient with the stomach polyp also had ileal congestion and edema and was diagnosed as having ileitis. A bulging lesion resembling a bowel segment protruding from the intestinal wall was detected at the jejunoileal junction in one patient, which was suspected to be intestinal duplication. In another patient, we found two openings in the lower ileum; the capsule passed through one opening, and the other opening was suspected to be a diverticulum (Table 1, Figure 2).

In patients with only abdominal pain, the diagnostic yield was 27.6%, while in patients with additional complaints, the diagnostic yield was 52.6%.

Of the six patients with abdominal pain and vomiting, three (50%) had endoscopic abnormalities, and two of these patients were finally diagnosed as having Crohn's disease and one was diagnosed as having congestive exudative gastritis. Of the seven patients with abdominal pain and gastrointestinal bleeding, five (71.2%) had endoscopic lesions (four with small intestinal ulcers and one with a diverticulum). In the three patients with abdominal pain accompanied by diarrhea and gastrointestinal bleeding, one patient was diagnosed as having Crohn's disease and one as having a duodenal ulcer.

### 3.5. Adverse Events

All patients successfully swallowed the capsule. Three patients required metoclopramide to help the capsule pass through the pylorus, and one also required the endoscopic delivery device. All patients felt that the MGCE procedure was comfortable and acceptable, and none had complaints during or after the examination. All patients were willing to undergo the procedure again. Of the 29 patients who had undergone traditional gastroscopy previously, all preferred MGCE. All patients excreted the capsule within 2 weeks.

## 4. Discussion

The Ankon MGCE system had a high diagnostic accuracy for gastric lesions compared with conventional gastroscopy, in several studies. Zou et al. [9] reported the first self-controlled comparative trial and showed a similar diagnostic accuracy for the two methods, with positive percent agreement, negative percent agreement, and overall agreement of 96.0%, 77.8%, and 91.2%, respectively. Using traditional gastroscopy as the gold standard, Liao et al. [7] found a sensitivity for MGCE of 90.4%, specificity of 94.7%, and diagnostic accuracy of 93.4%. Qian et al. [10] compared the ability of MGCE and gastroscopy to detect superficial gastric neoplasia and reported that the per-patient and per-lesion sensitivities of MGCE were 100% and 91.7%. High diagnostic accuracy allows us to use MGCE with confidence.

In addition to accuracy, another issue of concern is the cleanliness and visualization of the gastric cavity with MGCE. In our study, the cleanliness of all parts of the gastric cavity in most patients was sufficient to clearly evaluate the gastric cavity. In some patients, mucus, bubbles, food residue, and bile affected the cleanliness. However, by changing the patient's position, mucus floats to a different location and no longer blocks the capsule's camera; antifoaming agents help greatly for the bubbles [12]. Regarding visualization, we usually evaluate the gastric mucosa with patients in the left lateral, supine, and right lateral positions. Qian et al. [13] showed that a 5-position combination (left lateral, supine, right lateral, knee-chest, and sitting) had the highest rate of complete gastric landmark visualization. In our study, we added the sitting position, depending on the patient. In general, we had a better observation of the distal cavity than the proximal cavity, as both the cleanliness and the visualization was better in distally compared with proximally.

Traditional gastroscopy is considered to provide better evaluation of the duodenum because MGCE evaluates the duodenum passively. In our study, in 18.6% of patients, we pushed the capsule through the pylorus to explore the duodenum, but this is not done routinely. We believe that higher success rates for visualizing the duodenum are possible if we make the effort in every patient.

Compared with conventional gastroscopy, MGCE can avoid the discomfort caused by intubation and the adverse effects of narcotic drugs. In our study, all patients had a comfortable experience, and none experienced adverse events during follow-up. MGCE appears to be an excellent choice for pediatric patients, especially for those afraid of undergoing conventional gastroscopy.

In the pediatric patients with abdominal pain in our study, Crohn's disease was the most common diagnosis, followed by duodenal ulcers. Some studies [14–16] showed that capsule endoscopy in patients with the additional symptoms or signs, such as weight loss, increased the erythrocyte sedimentation rate, increased C-reactive protein level, and hypoalbuminemia, had a higher diagnostic yield. Egnatios et al. [17] suggested that capsule endoscopy in patients with nausea, weight loss, anemia, and a history of inflammatory bowel disease had a higher rate of abnormal findings, while May et al. [18] stated that the presence of additional symptoms did not increase the yield of abnormal findings. In our study, the diagnostic yield in patients with abdominal pain as the only symptom was lower than in patients with concurrent other symptoms, especially gastrointestinal bleeding, but we found no statistically significant difference. Most patients had undergone abdominal ultrasound, abdominal magnetic resonance imaging, colonoscopy, or other related examinations before being evaluated at our hospital, but without having the cause of their abdominal pain diagnosed. MGCE contributes to the diagnosis of pediatric patients with abdominal pain.

## 5. Conclusion

MGCE is safe, convenient, and tolerable for evaluating the gastric cavity and small intestine in pediatric patients. Overall, MGCE can effectively diagnose pediatric patients with abdominal pain.

## Figures and Tables

**Table 1:** Endoscopy findings and presumed diagnosis.

Location of findings	No	Endoscopy findings	No	Presumed diagnosis	No
Esophagus	1	Esophageal erosion	1	Reflux esophagitis	1

Stomach	3	Gastric polyp	1	Gastric polyps	1
		Gastric eminence lesion	1	Ectopic pancreas	1
		Gastric congestion and edema	1	Congestive exudative gastritis	1

Duodenum	4	Duodenal ulcer	4	Duodenal ulcer	4

Jejunoileum	11	Jejunal ulcer	2	Jejunal ulcer	1
		Ileal ulcer	2	Jejunoileal ulcers	1
		Ileal and jejunal ulcers	4	Crohn's disease	6
		Ileal congestion and edema	1	Ileitis	1
		Ileal diverticulum	1	Ileal diverticulum	1
		Small intestinal eminence lesions	1	Intestinal duplication	1

Total	19	Total	19	Total	19

## Data Availability

The data used to support the findings of this study are available from the corresponding authors upon request.
